# Enhanced Water Adsorption of MIL-101(Cr) by Metal-Organic Polyhedral Encapsulation for Adsorption Cooling

**DOI:** 10.3390/nano13071147

**Published:** 2023-03-23

**Authors:** Xiaoxiao Xia, Boyun Liu, Bo Zhao, Zichao Xia, Song Li

**Affiliations:** 1 School of Energy and Power Engineering, Huazhong University of Science and Technology, Wuhan 430074, China; 2 School of Power Engineering, Naval of University of Engineeing, Wuhan 430074, China

**Keywords:** adsorption cooling, water adsorption rate, encapsulation, composite, hydrophilicity

## Abstract

Metal-organic frameworks (MOFs) are one of the most promising adsorbents in the adsorption cooling system (ACS) for their outstanding water adsorption performance. Notwithstanding that fact, numerous reports pay more attention to the ACS performance improvement through enhancing equilibrium water uptake of MOFs. However, adsorption cooling performance, including specific cooling power (SCP) and coefficient of performance for cooling (COP_C_) of MOF/water working pairs, always depends on the water adsorption kinetics of MOFs in ACS. In this work, to increase the water adsorption rate, the preparation of MOP/MIL-101(Cr) was achieved by encapsulating hydrophilic metal-organic polyhedral (MOP) into MIL-101(Cr). It was found that the hydrophilicity of MOP/MIL-101(Cr) was enhanced upon hydrophilic MOP**3** encapsulation, resulting in a remarkable improvement in water adsorption rates. Furthermore, both SCP and COP_C_ for MOP/MIL-101(Cr)-water working pairs were also improved because of the fast water adsorption of MOP/MIL-101(Cr). In brief, an effective approach to enhance the water adsorption rate and cooling performance of MOF-water working pairs through enhancing the hydrophilicity of MOFs by encapsulating MOP into MOFs was reported in this work, which provides a new strategy for broadening the application of MOF composites in ACS.

## 1. Introduction

Increasing fuel consumption combined with anthropogenic greenhouse gas emissions are driving the escalating worldwide utilization of environmentally benign cooling technologies, especially renewable energy, to diminish global electrical energy requirements [1]. An adsorption cooling system (ACS) has been proposed, which enables the utilization of low-grade energy (e.g., solar radiation) for regeneration and water working fluid [2,3]. The efficiency indicators, including specific cooling power (SCP) and coefficient of performance for cooling (COP_C_), are used to quantify the cooling performance of ACS. Both SCP and COP_C_ are directly related to the water adsorption performance of adsorbents in terms of the level of mass exchange and the amount of heat required for regeneration [4,5]. Traditional porous adsorbents such as silica gels and zeolites are commonly employed in ACS. However, the unsatisfactory water adsorption performance and high regeneration temperatures limit their ACS performance [6,7,8]. Therefore, seeking high-performance adsorbents to enhance the adsorption cooling performance of the system has received growing attention.

The potential use of metal-organic frameworks (MOFs) [9] for ACS has recently received growing research interest [10] because of their superior water uptake [11]. MIL-101(Cr) [12] is one of the most prominent MOFs due to its ultrahigh water uptake (>1.0 g/g) and excellent chemical and structural stability in the water medium [13]. The equilibrium water uptake of MIL-101(Cr) (1.05 g/g [10]) is 3.5 times that of conventional silica gel (0.30 g/g [14]) at P = 2.85 kPa and T = 298 K. However, the superior equilibrium water uptake of the adsorbent does not always guarantee it outperforming SCP and COP_C_ in ACS since the slow water adsorption by adsorbents may greatly restrict their cyclic water uptake and, thus, cooling capacity [15,16]. For example, although the equilibrium water uptake by Al-fumarate (0.24 g/g at P = 1.2 kPa and T = 303 K) and FAM-Z01 (0.17 g/g at P = 1.2 kPa and T = 303 K) was lower than that by silica gel (0.47 g/g at P = 1.2 kPa and T = 303 K), their COP_C_ (COP_C_ = 0.41 for Al-fumarate and 0.47 for FAM-Z01) is higher than that of silica gel (COP_C_ = 0.26) owing to the faster water adsorption [15]. Moreover, the high equilibrium water uptake of adsorbents also does not guarantee fast adsorption kinetics [16]. It has been reported that the equilibrium water uptake of MIL-100(Fe) (0.26 g/g at P = 1.2 kPa and T = 303 K) is lower than that of MOF-801 (0.30 g/g at P = 1.2 kPa and T = 303 K) and the water adsorption of MIL-100(Fe) is 2.8 times faster than MOF-801 [16], indicating its high cooling power. Thus, the kinetic properties of adsorbents in water uptake play an important role in the cooling performance of ACS [17].

Regarding the factors influencing the water adsorption kinetics of MOFs, the hydrophilicity of MOFs accelerates water uptake. Recently, NH_2_-MIL-125 was reported to exhibit faster water adsorption than pristine MIL-125 owing to its improved hydrophilicity upon amine functionalization [18]. Meanwhile, OH-UiO-66 with hydroxy functionalization also exhibited enhanced hydrophilic properties, resulting in faster water adsorption than UiO-66 [19]. Although chemical functionalization is an effective strategy to enhance MOF hydrophilicity, it is complex and costly so it is not commonly used in the modification of MOFs [20,21]. The incorporation of functional guest species into MOFs [22] provides a facile strategy for fine-tuning the hydrophilicity of MOFs [23,24,25] under mild conditions [22]. Among these guest species, metal-organic polyhedral (MOP) [26] nanoparticles are a new class of inorganic-organic coordination complexes that can be prepared by the self-assembly of metal ions and organic ligands with hydrophilic functional groups [27]. However, agglomeration of MOP is unfavorable for its adsorption performance [27,28]. Thus, the encapsulation of MOP into MOFs was adopted [29], for example, by incorporating MOP-18 into IRMOF-74-IV [30]. Qiu et al. encapsulated M_6_L_4_ (a Pd-based MOP) into MIL-101(Cr) and revealed the improved catalytic performance of M_6_L_4_/MIL-101(Cr) composites [29]. The equilibrium water uptake of MOP/PCN-777 (0.26 g/g at P/P_0_ = 0.9 and T = 298 K) was remarkably higher than PCN-777 (0.16 g/g at P/P_0_ = 0.9 and T = 298 K), indicating the positive contribution of MOP to the water uptake of MOFs [28]. However, the influence of MOP encapsulation on the kinetic properties of MOFs for adsorption cooling has yet to be elucidated.

In this work, a Cu-based MOP named MOP**3** containing a hydrophilic −SO_3_H with a size of 2.9 nm [31] was chosen to tune the hydrophilicity of mesoporous MIL-101(Cr) with theoretical internal free diameters of 2.9 and 3.4 nm [12]. Structural characteristics and the water adsorption rates of MOP/MIL-101(Cr) with different MOP contents were measured. The adsorption cooling performance (SCP and COP_C_) for ACS based on MOP/MIL-101(Cr)-water working pairs were estimated using a lumped model-based mathematical modeling. The influence of MOP**3** on the structural characteristics, kinetic properties, and cooling performance of MOP/MIL-101(Cr) were investigated. The results provide insights into the exploration of MOP/MOF composites with enhanced kinetics properties by encapsulating MOP for high-performing adsorption cooling.

## 2. Materials and Methods

### 2.1. MOP/MIL-101(Cr) Preparation

**Figure 1:**
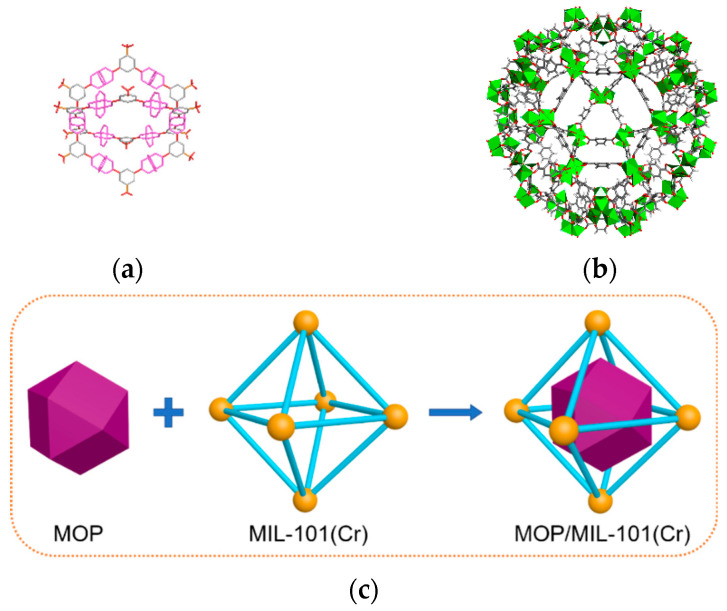
Structure of (**a**) MOP**3** and (**b**) MIL-101(Cr). (**c**) The schematic illustration of construction for MOP/MIL-101(Cr).

All chemicals and materials used for preparing MOP/MIL-101(Cr) composites were purchased from commercial sources without purification. MIL-101(Cr) [32] and MOP**3** [31] were synthesized via the previously reported method with details provided in the Supporting Information (SI). MOP/MIL-101(Cr) composites were prepared by a hydrophilicity-directed approach [29]; the construction diagram is shown in Figure 1. Specifically, MIL-101(Cr) was added to n-hexane with stirring. Then, Cu(NO_3_)_2_ and Na^+^SO_3_^−^-mBDC were dissolved in methanol and successively added to the suspension under continuous stirring. Afterward, 2,6-dimethylpyridine was added under stirring. Finally, the obtained solid was washed with DMF and deionized water and activated by heating under a vacuum. To avoid agglomeration, MOP**3** content in composites varies from 5 wt.% to 35 wt.%, corresponding to MOP/MIL-101(Cr)-*n* where *n* = 5, 10, 15, 20, and 35.

### 2.2. MOP/MIL-101(Cr) Characterization

The prepared MOP/MIL-101(Cr) composites were first characterized by powder X-ray diffraction (PXRD, PANalytical B.V., Netherlands) to confirm their crystalline structure. Then, scanning electron microscope (SEM, FEI, Netherlands) images were conducted to obtain the morphological characteristics of MOP/MIL-101(Cr) composites, from which particle size can be calculated by Image Pro Plus software. The Fourier transform infrared (FT-IR, Bruker, Germany) spectra were obtained to demonstrate MOP/MIL-101(Cr) composites’ structural characteristics and confirm MOP**3** encapsulation. The nitrogen (N_2_) adsorption isotherms were measured at 77 K (Quantachrome, US) to obtain the BET surface area, total pore volume, and pore size distribution of MOP/MIL-101(Cr) composites. The porosity of MOP/MIL-101(Cr) composites and MIL-101(Cr) were calculated using true density determined by a true density analyzer (Micromeritics, US) and bulk density based on volume measured in a container of a known volume. Differential scanning calorimeter (DSC, PerkinElmer, US) was conducted under N_2_ atmosphere to obtain the heat capacity of MOP/MIL-101(Cr) composites and MIL-101(Cr). Water adsorption isotherms were measured at 288, 298, and 303 K at relative pressure ranges from 0.001 to 0.9 (Quantachrome, US). The data were used to calculate the heat of adsorption of MOP/MIL-101(Cr) composites and MIL-101(Cr) using the Clausius–Clapeyron equation.

### 2.3. Water Adsorption Rate Measurement

Generally, the adsorption temperature and humidity were 303 K and 30% (RH) in the adsorption bed under typical cooling conditions for the ACS [33]. Thus, the dynamic water adsorption curve (Surface Measurement Systems, UK) at 303 K and 30% relative humidity was tested with the details provided in the SI, from which the water adsorption rate of MOP/MIL-101(Cr) composites and MIL-101(Cr) can be calculated by differentiating the water uptake from the corresponding adsorption time. For comparison, the average water adsorption rate was obtained by dividing water uptake by adsorption time.

### 2.4. Mathematical Modeling of ACS

**Figure 2:**
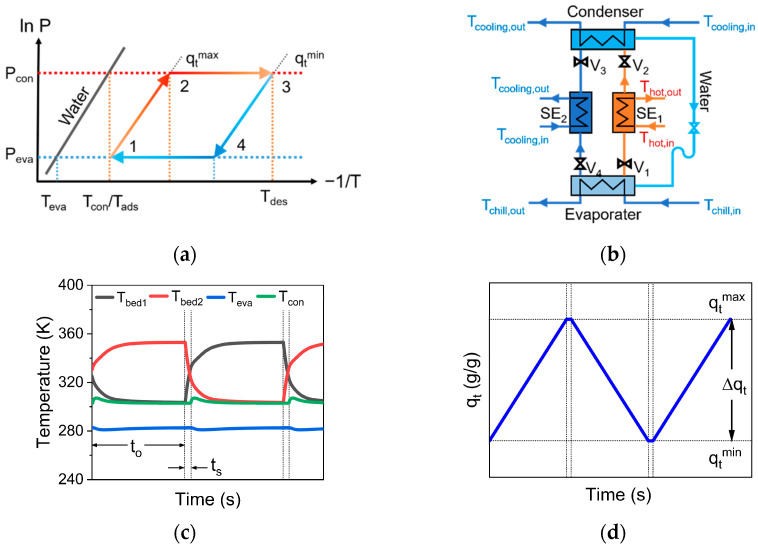
Schematic diagram of (**a**) thermal dynamic cycle, (**b**) two-bed adsorption cooling system, (**c**) transient temperature, and (**d**) transient water uptake in ACS.

A dynamic thermal cycle of ACS (Figure 2a) includes pre-heating (from 1 to 2), desorption (from 2 to 3), pre-cooling (from 3 to 4), and adsorption (from 4 to 1). In order to explore the adsorption cooling performance of MOP/MIL-101(Cr)-water working pairs, a typical two-bed adsorption cooling system [34] was used in this work. The two-bed ACS includes a condenser, an evaporator, two adsorption beds, and several circulation pipes, including hot water, cooling water, and chilled water circulation pipes (Figure 2b).

As shown in Figure 2b, adsorbents in SE_1_ (desorption bed) are desorbed or regenerated (T_des_) by an external heat source (Q_regen_). Meanwhile, SE_1_ is connected with the condenser (T_con_ and P_con_). Thereby, the desorbed water (gaseous) enters the condenser from SE_1_ and condenses into liquid water. Subsequently, the condensed water flows into the evaporator and evaporates into water vapor (T_eva_ and P_eva_). At the same time, the outside heat can be taken away by water evaporation (Q_eva_), thus producing a cooling effect. Then, the evaporated water vapor enters SE_2_ (adsorption bed) from the evaporator for adsorption. The schematic diagram of the transient temperature (Figure 2c) and of the water uptake (Figure 2d) for two-bed ACS is shown in Figure 2. The system follows the mass and energy balance equations. The energy balance equation for the adsorption bed can be described as:

(1) $$\left(M_{ad}C_{p,ad}+q_{bed}M_{ad}C_{p,w}+M_{hex}C_{p,hex}\right)\frac{dT_{bed}}{dt}=\phi M_{ad}q_{st}\frac{dq_{bed}}{dt}-\left(m_{bed,w}C_{p,w}\right)\left(T_{bed,out}-T_{bed,in}\right)$$

Here, items on the left successively represent the change in thermal energy of adsorbents (MOP/MIL-101(Cr)), the working fluid (water), and the heat exchanger. Items on the right represent the heat of adsorption in the adsorption bed and heat transfer between the adsorption bed and evaporator. ϕ = 0 at switch time (t_s_) and ϕ = 1 at operating time (t_o_). Heat capacity (C_p,ad_) and heat of adsorption (q_st_) for MOP/MIL-101(Cr) are shown in Table 1. The energy balance equations in the evaporator and condenser are shown in SI.

The mass balance equation in ACS:

(2) $$\frac{dM_{w}}{dt}=-M_{ad}\left(\frac{dq_{ads}}{dt}+\frac{dq_{des}}{dt}\right)$$

The rate for the change in water mass is equal to the amount of adsorbed water during adsorption and how much water was desorbed during desorption. The equation parameters are shown in Table S1 [35].

Based on the energy and mass balance equations in ACS, SCP, and COP_C_ of MOP/MIL-101(Cr)-water working pairs are calculated as follows:

(3) $$SCP=\frac{Q_{eva}}{t_{cycle}}$$

(4) $$COP_{C}=\frac{Q_{eva}}{Q_{regen}}$$

Here, Q_eva_ and Q_regen_ are the cooling capacity and total energy consumption of MOP/MIL-101(Cr) in ACS; t_cycle_ is the cycle time of ACS.

The adsorption temperature (T_ads_) of ACS is equal to the condensation temperature (T_con_) [36], which is usually 303 K in cooling conditions (Table 2). According to the hot water temperature generated by solar energy, the desorption temperature (T_des_) is set at 353 K [37]. The chilled water inlet temperature determines the evaporation temperature (T_eva_), which is generally set to 283 K for cooling. Moreover, mass flow in circulation pipes, including hot water (m_hot_), cooling water (m_cooling_), and chilled water (m_chill_); the cycle time, including operating time (t_o_) and switch time (t_s_) are set based on previous reports [34,35,38] (Table 2).

## 3. Results and Discussion

### 3.1. Effects of Structural Characteristics on Water Adsorption Rates

**Figure 3:**
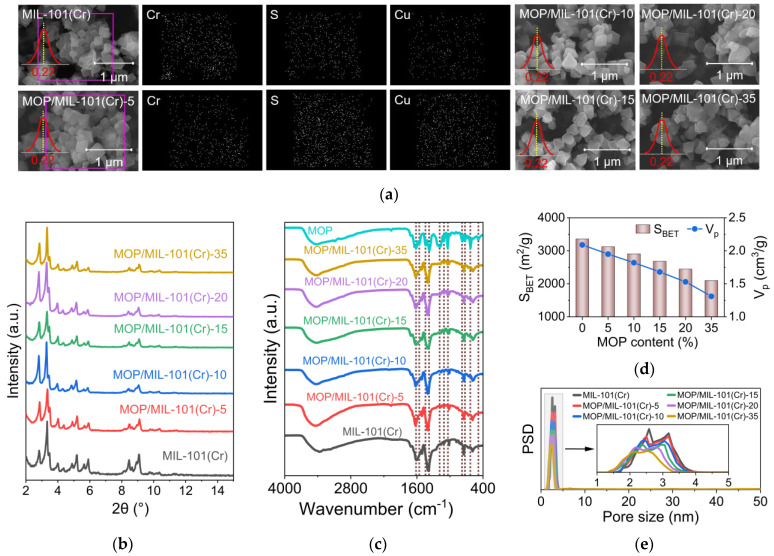
Structural characterization of MOP/MIL-101(Cr) composites. (**a**) SEM images, (**b**) PXRD patterns, (**c**) FT-IR spectra, (**d**) BET surface areas (S_BET_) and total pore volumes (V_p_), (**e**) Pore size distributions (PSD).

A previous study showed that the structural properties of MOFs will affect their water adsorption performance. For example, the large pore volume is favorable for the equilibrium water uptake of MOFs [11]. Therefore, we first characterized the structural properties of MOP/MIL-101(Cr). Scanning electron microscopy (SEM) images demonstrated that the morphology of MOP/MIL-101(Cr) composites was not significantly changed compared with pristine MIL-101(Cr) (Figure 3a). The homogeneous distribution of Cu element in MOP/MIL-101(Cr)-5 was observed by energy dispersive spectroscopy elementary mapping (Figure 3a). In contrast, no significant Cu signal in MIL-101(Cr) indicates the uniform distribution of MOP**3** in composites. In addition, compared to MIL-101(Cr), the particle size of MOP/MIL-101(Cr) composites was not changed after MOP**3** incorporation, regardless of MOP**3** content. Moreover, the original crystallinity of MIL-101(Cr) was effectively preserved as demonstrated in powder X-ray diffraction (PXRD) patterns of MOP/MIL-101(Cr), which matches with MIL-101(Cr) (Figure 3b).

The successful encapsulation of MOP**3** in MIL-101(Cr) was confirmed by Fourier transform infrared (FT-IR) spectra (Figure 3c). The peak at 1608 cm^−1^ represents the asymmetric stretching pattern of the COO^−^-Cu^2+^ complex [39]. The peak at 1042 cm^−1^ results from the symmetric stretching pattern of the SO^3−^ [28] present in MOP**3**. The results are consistent with previous reports on the encapsulation of MOP**3** into PCN-777 [28]. Although the morphology and crystallinity of MIL-101(Cr) are well preserved after MOP**3** incorporation, BET surface area and total pore volume of MOP/MIL-101(Cr) decrease significantly upon MOP**3** encapsulation (Figure 3d). Compared with MIL-101(Cr), the BET surface area and total pore volume of MOP/MIL-101(Cr)-35 decreased by 37.5% and 37.3%, respectively. In addition, the pore size of MOP/MIL-101(Cr) is gradually reduced with the increased loading of MOP**3** (Figure 3e), which was also observed in a previous study on MOP/PCN-777 composites [28].

**Figure 4:**
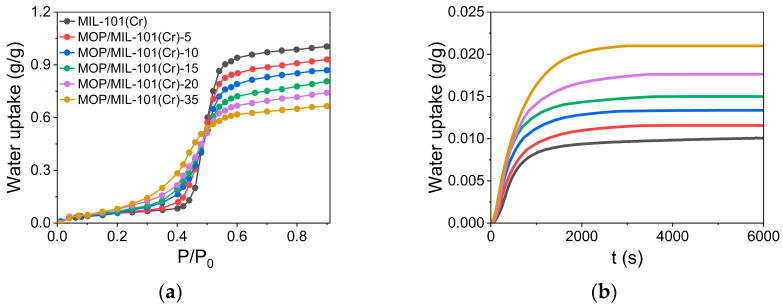
(**a**) Water adsorption isotherms of MOP/MIL-101(Cr) composites at 303 K with the relative pressure ranges from 0.001 to 0.9. (**b**) Dynamic water adsorption curves of MOP/MIL-101(Cr) composites at 303 K and 30% relative humidity.

Furthermore, the BET surface area and total pore volume are key factors affecting the equilibrium water uptake of MOFs under high pressure [11]. Thus, the reduction in BET surface area and total pore volume leads to a decrease in equilibrium water uptake for MOP/MIL-101(Cr) at a high relative pressure of P/P_0_ > 0.5 (Figure 4a). However, the equilibrium water uptake of MOP/MIL-101(Cr) increased at low pressure of P/P_0_ < 0.5, ascribed to enhanced hydrophilicity of MIL-101(Cr) upon hydrophilic MOP**3** encapsulation. Herein, Henry’s constant (K_H_), which describes the affinity of adsorbents toward the water at low pressure [40], was used to quantify the hydrophilicity of MOP/MIL-101(Cr) composites. It was found that the hydrophilicity of MOP/MIL-101(Cr) gradually increased with the MOP**3** content. To investigate the reason for the enhanced hydrophilicity of MOP/MIL-101(Cr), K_H_ of MOP**3** and MIL-101(Cr) were measured (Figure S1). K_H_ of MOP**3** (K_H_ = 0.116 g/g·kPa) is higher than MOP/MIL-101(Cr) (K_H_ = 0.095 ~ 0.110 g/g·kPa) and MIL-101(Cr) (K_H_ = 0.095 g/g·kPa), indicating the enhanced hydrophilicity of MOP/MIL-101(Cr) resulting from MOP**3** encapsulation. According to previous reports, hydrophilicity is beneficial for enhancing water adsorption rate [18,19]. Results show that water adsorption rates of MIL-101(Cr) are significantly increased upon MOP**3** encapsulation. In addition, water adsorption rates of MOP/MIL-101(Cr) are increased linearly with MOP**3** content, consistent with Henry’s constant.

**Figure 5:**
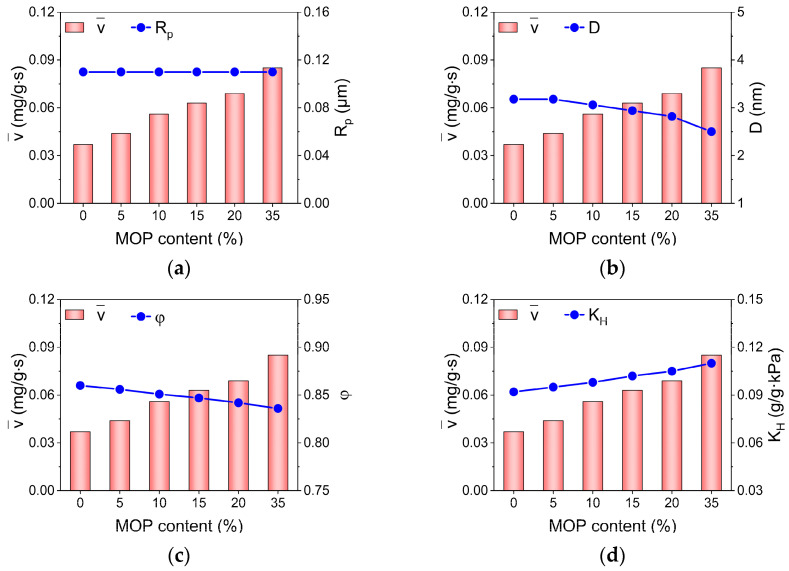
Relationship between (**a**) particle size (R_p_), (**b**) pore size (D), (**c**) porosity (φ), (**d**) Henry’s constant (K_H_), and water adsorption rate ($\bar{v}$) of MOP/MIL-101(Cr) composites with different MOP**3** content.

Moreover, apart from surface hydrophilicity, other structure properties including pore size, particle size and porosity may also impact on water adsorption kinetics. Based on diffusion theory, Knudsen diffusion and molecular diffusion dominate water adsorption in micropores and mesopores, respectively. Thus, either the pores within sample particles or the interparticle pores may impose impacts on water adsorption kinetics. In addition, porosity is frequently used to represent the structural characteristics of porous media. To explore the correlation between structural properties of MOP/MIL-101(Cr) and water adsorption rate, the impacts of particle size (R_p_), pore size (D), porosity (φ), and hydrophilicity (K_H_) on the water adsorption rate ($\bar{v}$) of MOP/MIL-101(Cr) was investigated (Figure 5). In theory, larger particle/pore size and high porosity are beneficial for water diffusion in pore media [41]. However, in this work, both pore size and porosity were reduced with increased MOP**3** content, and the particle size remained nearly constant. These outcomes are not conducive to improving the water adsorption rate for MOP/MIL-101(Cr). Nevertheless, the water adsorption rates of MOP/MIL-101(Cr) exhibited a consistent tendency to hydrophilicity with the increase in MOP**3** content. This suggests the dominant role of hydrophilicity in water adsorption rates of MOP/MIL-101(Cr). MOP/MIL-101(Cr)-35 exhibited the highest water adsorption rate, which was approximately 2.3 times that of MIL-101(Cr).

### 3.2. Adsorption Cooling Performance

**Figure 6:**
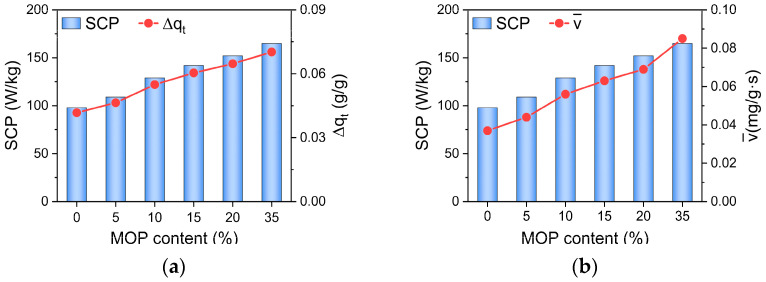
Relationship between (**a**) cyclic working capacity (Δq_t_), (**b**) water adsorption rate ($\bar{v}$), and SCP of MOP/MIL-101(Cr)-water working pairs at different MOP**3** content.

Cooling performance, including SCP and COP_C_ of MOP/MIL-101(Cr)-water working pairs, were subsequently discussed. As shown in Figure 6, the SCP of MOP/MIL-101(Cr)-water increases with the MOP**3** content. SCP measures the capacity of the system to produce a cooling effect [42], mainly determined by cooling capacity (Q_eva_) based on Equation (3). Furthermore, the cooling capacity (Q_eva_) is mainly affected by cyclic working capacity (Δq_t_) according to Q_eva_ = L_H_∙Δq_t_, where L_H_ represents the latent heat of water vaporization, which is a constant at fixed evaporation temperature. Δq_t_ can be calculated by transient water uptake (Figure 2d). Therefore, the large cyclic working capacity of MOP/MIL-101(Cr) is favorable for SCP of MOP/MIL-101(Cr)-water (Figure 6a). Notably, the cyclic working capacity of MOP/MIL-101(Cr) improves with the increase in MOP**3** content, which exhibits a consistent tendency with water adsorption rate (Figure 6). This indicates that the cyclic working capacity is mainly affected by the water adsorption rate of MOP/MIL-101(Cr). Consequently, MOP/MIL-101(Cr) with fast water uptake promotes the cyclic working capacity resulting in the enhancement of SCP. Moreover, compared to MIL-101(Cr)-water, SCP of MOP/MIL-101(Cr)-water enhanced regardless of MOP**3** content because of the fast water adsorption of MOP/MIL-101(Cr).

The COP_C_ is defined as the ratio of the useful energy provided by the device and the required input [43]

$$COP_{C}=\frac{SCP\cdot t_{cycle}}{Q_{regen}}$$

**Figure 7:**
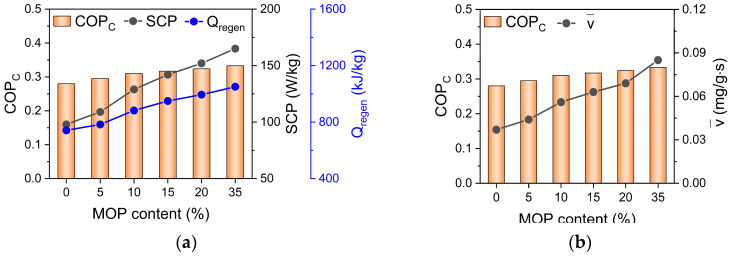
Relationship between (**a**) SCP, total energy consumption (Q_regen_), (**b**) water adsorption rate ($\bar{v}$), and COP_C_ of MOP/MIL-101(Cr)-water working pairs at different MOP**3** content.

COP_C_ of MOP/MIL-101(Cr)-water increases with MOP**3** content, which exhibited a consistent tendency with total energy consumption of system (Q_regen_) and SCP (Figure 7a) as well as water adsorption rate of MOP/MIL-101(Cr) (Figure 7b). The above analysis suggests that fast water adsorption of MOP/MIL-101(Cr) can promote SCP. Subsequently, we further investigated the total energy consumption (Q_regen_) of MOP/MIL-101(Cr).

**Figure 8:**
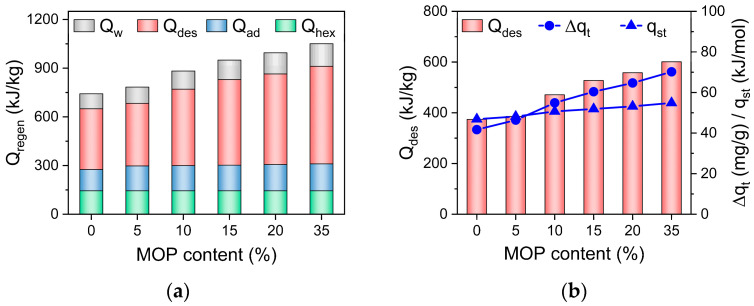
(**a**) Energy consumption for temperature rising of adsorbents (Q_ad_), adsorbed water (Q_w_), and heat exchanger (Q_hex_) during the desorption process and energy consumption for water desorption (Q_des_). (**b**) Relationship between cyclic working capacity (Δq_t_), the heat of adsorption (q_st_), and Q_des_ of MOP/MIL-101(Cr) composites with different MOP**3** content.

Total energy consumption (Q_regen_ = Q_ad_ + Q_w_ + Q_hex_ + Q_des_) of ACS is composed of four parts based on the energy balance in the adsorption bed (Equation (1)): energy consumption by temperature rising (from adsorption temperature to desorption temperature) of adsorbents or sensible heat of adsorbents (Q_ad_ = C_p,ad_(T_des_ − T_ads_)), the sensible heat of adsorbed water ($Q_{w}=\bar{v}\cdot t_{o}\cdot C_{p,w}\left(T_{des}-T_{ads}\right)$) and the sensible heat of heat exchanger ($Q_{hex}=\frac{M_{hex}}{M_{ad}}C_{p,hex}\left(T_{des}-T_{ads}\right)$) during desorption process, and energy consumption for water desorption (Q_des_ = Δq_t_∙q_st_). It is noted (Figure 8) that the Q_hex_ is a constant at the fixed temperature and mass of adsorbents. The sensible heat of MOP/MIL-101(Cr) (Q_ad_) and adsorbed water (Q_w_) are mainly affected by the heat capacity of MOP/MIL-101(Cr) (C_p,ad_) and water adsorption rate, respectively. The slight increase in Q_ad_ with the MOP**3** content can be ascribed to the increased heat capacity of MOP/MIL-101(Cr) upon MOP**3** (C_p,ad_ = 1.77 J/g·K for MOP**3**, Table 1). Q_w_ was also slightly improved because of the increased water adsorption rate of MOP/MIL-101(Cr) with MOP**3** content. In contrast, the energy consumption of water desorption (Q_des_) mostly contributes to total energy consumption (Figure 8a), which is ascribed to the significantly increased hydrophilicity of MOP/MIL-101(Cr) upon MOP**3** encapsulation that requires the increased energy consumption for desorption. In addition, the increased heat of adsorption (q_st_, Table 1) and cyclic working capacity of MOP/MIL-101(Cr) resulting from fast water adsorption also favor the remarkable increase in Q_des_ (Figure 8b). In conclusion, the improved water adsorption rate promotes SCP of MOP/MIL-101(Cr)-water working pairs, resulting in the significant enhancement of SCP. Energy consumption of MOP/MIL-101(Cr) can be enhanced by increased cyclic working capacity and heat of adsorption, which is not beneficial for improving COP_C_. Nonetheless, the improved SCP caused by accelerated water adsorption contributes to the enhancement of COP_C_. Thus, the COP_C_ of MOP/MIL-101(Cr)-water working pairs increased with MOP**3** content.

## 4. Conclusions

In this work, we report on a new strategy to enhance the water adsorption kinetics and cooling performance of MIL-101(Cr) and its working pair by encapsulating MOP**3** into MIL-101(Cr). We prepared and characterized MOP/MIL-101(Cr) composites with different MOP**3** contents and found that the encapsulation of MOP**3** decreased the pore size and porosity of MIL-101(Cr), while the hydrophilicity of MOP/MIL-101(Cr) significantly enhanced with the increase in MOP**3** content. Although reduced pore size and porosity are detrimental to water adsorption, the water adsorption rate of MOP/MIL-101(Cr) improved with the increase in MOP**3** due to the enhancement of hydrophilicity. Based on mathematical modeling, the adsorption cooling performance, namely both SCP and COP_C_, of MOP/MIL-101(Cr)-water for ACS increases with the MOP**3** content. This can be ascribed to the increased water adsorption rate resulting from the improved hydrophilicity of MOP/MIL-101(Cr) upon MOP**3** encapsulation. These findings demonstrate the effectiveness of MOP**3** encapsulation in improving the water adsorption kinetics and cooling performance of MIL-101(Cr).

## Figures and Tables

**Table 1:** Heat capacity (C_p,ad_) and heat of adsorption (q_st_) of MOP/MIL-101(Cr).

Adsorbents	C_p,ad_ (kJ/K∙kg)	q_st_ (kJ/kg)
MIL-101(Cr)	1.31	2603
MOP/MIL-101(Cr)-5	1.53	2676
MOP/MIL-101(Cr)-10	1.55	2815
MOP/MIL-101(Cr)-15	1.58	2883
MOP/MIL-101(Cr)-20	1.62	2954
MOP/MIL-101(Cr)-35	1.66	3048

**Table 2:** Temperature, mass flow, and cycle time of ACS.

Parameters	Symbol	Value
Adsorption temperature	T_ads_	303 K
Evaporation temperature	T_eva_	283 K
Condensation temperature	T_con_	303 K
Desorption temperature	T_des_	353 K
Hot water mass flow	m_hot_	1.5 kg/s
Cooling water mass flow	m_cooling_	1.5 kg/s
Chilled water mass flow	m_chill_	1.0 kg/s
Operating time	t_o_	1000 s
Switch time	t_s_	60 s

## Data Availability

Not applicable.

## Supplementary Materials

The following supporting information can be downloaded at: https://www.mdpi.com/article/10.3390/nano13071147/s1, Figure S1: (**a**) water adsorption isotherms and (**b**) K_H_ of MOP/MIL-101(Cr), MIL-101(Cr) and MOP**3**; Table S1: parameters of ACS; Table S2: fitting parameters of the universal adsorption isotherm model; Table S3: fitting parameters of modified linear driving force model.
