# Supplementary material for: Enhanced Water Adsorption of MIL-101(Cr) by Metal-Organic Polyhedral Encapsulation for Adsorption Cooling
Source: Nanomaterials (Basel). 2023 Mar 23;13(7):1147. doi: 10.3390/nano13071147 (PMC10096998; doi:10.3390/nano13071147)
Supplement: Supplementary file 1 [file nanomaterials-13-01147-s001.zip › nanomaterials-2291093-supplementary.pdf]

Supporting Information

Enhanced Water Adsorption of MIL-101(Cr) by Metal-Organic Polyhedral Encapsulation for Adsorption Cooling

Materials. All chemicals and materials were used as received (without any purification) from commercial sources. Chromium nitrate nonahydrate ($Cr(NO_3)_3·9H_2O$, 99.0%), terephthalic acid (H_2BDC, 99.0%), N,N-Dimethylacetamide (DMA, 99.8%), copper acetate monohydrate ($Cu(CH_3COO)_2·H_2O$, 98.0%), 5-Sulfoisophthalic acid sodium salt (Na^+SO3^--mBDC, 98.0%), 2,6-dimethylpyridine (99.0%), n-hexane (98.0%) and copper nitrate trihydrate (Cu(NO3)2·3H2O, 99%) were purchased from Shanghai Aladdin Bio-Chem Technology Co. Ltd. Glacial acetic acid, N,N-dimethylformamide (DMF), ethanol, methanol (MeOH), N,N-Dimethylacetamide (DMA) and nitric acid were provided by Sinopharm Chemical Reagent Co. Ltd. (Shanghai, China, AR). Nitrogen (N_2, 99.99%) and helium (He, 99.999%) gases were purchased from Wuhan Huaerwen Co.

MIL-101(Cr) preparation [1]. $Cr(NO_3)_3·9H_2O$ (4.00 g, 10 mmol) and H_2BDC (1.66 g, 10 mmol) were added in deionized water (50 mL) to obtain a mixture. Glacial acetic acid (0.58 mL) was added into the mixture. After that, the mixture was sonicated for 30 minutes at room temperature. Then, the mixture was transferred into a 100 mL capacity Teflon-lined autoclave and heated at 493 K for 8 h. After cooling to room temperature, the green solids were washed successively with deionized water, DMF and ethanol (30 mL×3). The obtained solids were dried overnight at 423 K under vacuum.

MOP3 preparation [2]. Dissolved Na^+SO3^--mBDC (0.67 g, 2.5 mmol) in MeOH (20 mL), and dissolved $Cu(CH_3COO)_2·H_2O$ (0.5 g, 2.5 mmol) in MeOH/DMA (37.5 mL, v/v = 1:1). Then, mixed the above two solutions. After 20 minutes, DMA (12.5 mL) and nitric acid (0.1 mL) were added with

vial opened at room temperature. After 10 days, synthesized blue particles were collected and washed with DMA.

Characterization. All the adsorbents were characterized by powder X-ray diffraction (PXRD) to confirm their crystalline structure under room temperature and air environment. A X'Pert PRO MPD X-ray diffractometer from PANalytical B.V. (Netherlands) with Cu Kα radiation (λ=1.540598 Å) was used. The 2θ range from 2° to 15° with a continue step size of 0.016711° and scan rate of 0.12 s per step. Scanning electron microscope (SEM) images were conducted using a Nova NanoSEM 450 SEM instrument from FEI (Netherlands) on the samples previously dried and sputter-coated with a gold layer at an accelerating voltage of 30 kV for 12 min. The crystal size was calculated by SEM images. The Fourier transform infrared (FT-IR) spectra were obtained using a VERTEX 70 FT-IR spectrometers from Bruker (Germany) to get the structural characteristic of adsorbents. The data collected in the spectral range of 4000 ~ 400 cm^{-1}.The nitrogen (N_2) adsorption isotherms were measured at 77 K on a Quantachrome Autosorb-iQ2 gas analyzer (US). After vacuum degassing at 393 K for 24 h before measurement, the sample tube was put into the cold well of 77 K and a fixed amount of N_2 was filled. The Brunauer-Emmett-Teller (BET) surface area was determined with a relative pressure (P/P_0) range of 0.08 ~ 0.25 on N_2 adsorption isotherm based on the BET theory, and the total pore volume was determined at $P/P_0 = 0.99$. Pore size distribution was obtained by using non-local density functional theory methods. The true density was confirmed by a AccuPyc II 1340 instrument from Micromeritics (US). The mass of samples can be measured in a container with certain volume to get bulk density. Then, the porosity was obtained from the true density and bulk density. Heat capacity was measured with Diamond DSC differential scanning calorimeter from PerkinElmer (US) at 273 ~ 373 K with gas atmosphere as nitrogen. The heating rate is 1 K/min and 20 mg MOP/MIL-101(Cr) composites are used in DSC measurement. Heat capacity of MOP/MIL-101(Cr) composites can be calculated by comparing them with the standard sample under the same heating rate. Heat of adsorption

was calculated by Clausius-Clapeyron equation ($q_{st} = -R\frac{\partial(\ln P)}{\partial(1/T)}$) with adsorption isotherms at different working temperature.

Mathematical modeling of ACS. The outlet temperature of adsorption bed can be expressed as a logarithmic mean temperature difference method as:

$$T_{bed,out}=T_{bed}+\left(T_{bed,in}-T_{bed}\right)\exp\left(-\frac{U_{bed}A_{bed}}{m_{bed,w}C_{p,w}}\right)$$

The outlet temperature of the condenser can be expressed by the logarithmic mean temperature difference method as:

$$T_{cooling,out}=T_{con}+\left(T_{cooling,in}-T_{con}\right)\exp\left(-\frac{U_{con}A_{con}}{m_{cooling,w}C_{p,w}}\right)$$

The outlet temperature of the evaporator can be expressed by the logarithmic mean temperature difference method as:

$$T_{chill,out}=T_{eva}+\left(T_{chill,in}-T_{eva}\right)\exp\left(-\frac{U_{eva}A_{eva}}{m_{chill,w}C_{p,w}}\right)$$

The parameters in equations are shown in Table S1 [3].

Table S1. Parameters of adsorption cooling system.

Parameter	Description	Value
M_{ad}	Mass of adsorbent	60 kg
$(M\cdot C_p)_{bed}$	Thermal mass of adsorption bed	86.7 kJ/K

$(M \cdot C_p)_{con}$	Thermal mass of condenser	$9.341+5\times C_{p,wf}$ kJ/K
$(M \cdot C_p)_{eva}$	Thermal mass of evaporator	$4.825+80\times C_{p,wf}$ kJ/K
$U_{bed}A_{bed}$	Total heat transfer coefficient of adsorption bed	2.952 kW/K
$U_{con}A_{con}$	Total heat transfer coefficient of condenser	15.33 kW/K
$U_{eva}A_{eva}$	Total heat transfer coefficient of evaporator	4.87 kW/K

Table S2. Fitting parameters of the universal adsorption isotherm model.

Adsorbent	n	μ_i	ε_{oi}（J/mol）	m_i（J/mol）
MIL-101(Cr)	3	$\mu_1=0.85$	$\varepsilon_{o1}=1.86\times10^3$	$m_1=6.61\times10^1$
		$\mu_2=0.14$	$\varepsilon_{o2}=2.87\times10^3$	$m_2=2.67\times10^3$
		$\mu_3=0.01$	$\varepsilon_{o3}=9.49\times10^3$	$m_3=1.38\times10^2$
MOP/MIL-101(Cr)-5	3	$\mu_1=0.85$	$\varepsilon_{o1}=1.80\times10^3$	$m_1=1.66\times10^2$
		$\mu_2=0.02$	$\varepsilon_{o2}=1.41\times10^5$	$m_2=1.98\times10^2$
		$\mu_3=0.13$	$\varepsilon_{o3}=2.72\times10^3$	$m_3=1.61\times10^3$
MOP/MIL-101(Cr)-10	3	$\mu_1=0.80$	$\varepsilon_{o1}=1.94\times10^3$	$m_1=2.66\times10^2$
		$\mu_2=0.09$	$\varepsilon_{o2}=4.96\times10^3$	$m_2=3.50\times10^3$
		$\mu_3=0.11$	$\varepsilon_{o3}=1.81\times10^3$	$m_3=3.68\times10^1$
MOP/MIL-101(Cr)-15	3	$\mu_1=0.50$	$\varepsilon_{o1}=1.88\times10^3$	$m_1=1.41\times10^2$
		$\mu_2=0.06$	$\varepsilon_{o2}=6.43\times10^3$	$m_2=1.44\times10^3$
		$\mu_3=0.44$	$\varepsilon_{o3}=2.00\times10^3$	$m_3=6.27\times10^2$
MOP/MIL-101(Cr)-20	3	$\mu_1=0.51$	$\varepsilon_{o1}=2.00\times10^3$	$m_1=5.36\times10^2$
		$\mu_2=0.09$	$\varepsilon_{o2}=5.86\times10^3$	$m_2=1.40\times10^3$
		$\mu_3=0.40$	$\varepsilon_{o3}=1.91\times10^3$	$m_3=1.29\times10^2$
MOP/MIL-101(Cr)-35	3	$\mu_1=0.72$	$\varepsilon_{o1}=2.13\times10^3$	$m_1=2.84\times10^2$

	μ_2=0.07	ε_{o2}=1.99×10^3	m_2=8.55×10^1
	μ_3=0.21	ε_{o3}=3.88×10^3	m_3=1.52×10^3

Table S3. Fitting parameters of modified linear driving force model.

Adsorbent	κ	β
MIL-101(Cr)	4.604×10^{-4}	0.696
MOP/MIL-101(Cr)-5	1.386×10^{-3}	0.897
MOP/MIL-101(Cr)-10	2.027×10^{-3}	1.138
MOP/MIL-101(Cr)-15	2.303×10^{-3}	1.402
MOP/MIL-101(Cr)-20	2.523×10^{-3}	1.405
MOP/MIL-101(Cr)-35	2.913×10^{-3}	1.512

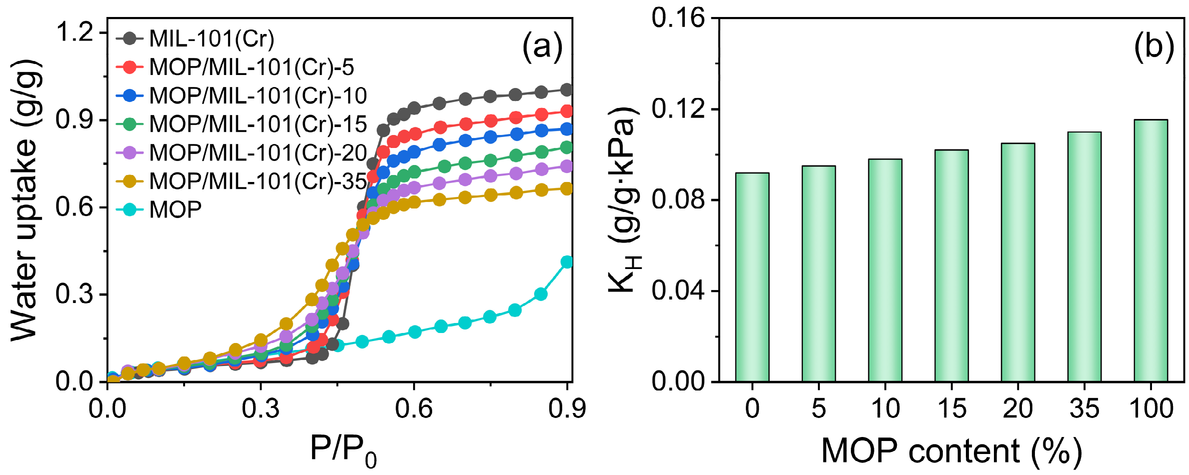

Figure S1. (a) Water adsorption isotherms and (b) K_H of MOP/MIL-101(Cr), MIL-101(Cr) and MOP.

Reference

1. Rallapalli, P.B.S.; Raj, M.C.; Senthilkumar, S.; Somani, R.S.; Bajaj, H.C. HF-Free Synthesis of MIL-101(Cr) and Its Hydrogen Adsorption Studies. *Environ. Prog. Sustain.* **2016**, *35*, 461–

468.

2. Lee, J.; Lim, D.W.; Dekura, S.; Kitagawa, H.; Choe, W. MOP × MOF: Collaborative Combination of Metal-Organic Polyhedra and Metal-Organic Framework for Proton Conductivity. *ACS Appl. Mater. Interfaces* **2019**, *11*, 12639–12646.

3. Saha, B.B.; El-Sharkawy, I.I.; Chakraborty, A.; Koyama, S. Study on an Activated Carbon Fiber-Ethanol Adsorption Chiller: Part I – System Description and Modelling. *Int. J. Refrig.* **2007**, *30*, 86–95.
